# Impact of excessive interest in news related to the war on mental health in conditions of big invasion and information war: experience of Ukraine

**DOI:** 10.1192/j.eurpsy.2024.1388

**Published:** 2024-08-27

**Authors:** M. Markova, T. Aliieva, T. Abdriakhimova, H. Prib, A. Markov

**Affiliations:** ^1^Kharkiv National Medical University, Kharkiv; ^2^ Bogomolets National Medical University; ^3^Academy of Labour, Social Relations and Tourism, Kyiv, Ukraine

## Abstract

**Introduction:**

An important component of the russian-Ukrainian war is information war. russia conducts psychological diversions the purpose of which is harming and disorganization the population’s mental health by reducing the ability of criticality thinking and formation of various stress-associated, anxiety-depressive, phobic, etc. disorders.

**Objectives:**

To study the impact of information about the war on the mental health of Ukrainian population.

**Methods:**

186 Ukrainian people voluntarily completed the questionnaire in Google format. It contains tools for assessing the level of stress PTCI, anxiety response GAD-7, depression PHQ-9, the intolerance to the uncertainty (IUS-12 in G.Gromova’ adaptation) and developed by us “Test for the detection of disorders related to the obsession with news about the russian-Ukrainian war” (M.Markova et al, 2022).

**Results:**

Most of the civilian population of Ukraine demonstrate excessive fascination with news associated with the war, with almost 50% have all the signs of clinically formed addiction.

Psychopathological anxiety and depressive manifestations of varying intensity are characterized by more than 30% of the population. Almost 80% suffer from the effects of psycho-traumatic factors, of which 45% are observed by post-stress maladaptation, 25% - by signs of PTSD (23%) or PTSD (2%).

The presence of anxiety-depressive response does not depend on the level of obsession of the news: among persons with signs and/or clinical psychopathological symptoms, there are persons both excessively passionate about information and with a safe level of use.

Any high level of interest of news (addiction/dangerous/risky) has a close direct correlation with the intensity of pathological stressful response and the level of tolerance to uncertainty.

All persons with anxiety-depressive and pathological stressful response symptoms, and 58% of people with excessive fascination with news are characterized by low tolerance to uncertainty. This can serve as a prognostic marker of development of maladaptation and testify to the leading role of a lack of tolerance to uncertainty in its development in wartime.

42 % of people who have an excessive interest in news, haven’t signs of maladaptive response. They use the interest of information as a stress management’s resource, which has a positive effect on the mental state and increases the ability to successfully function in uncertainty.

**Conclusions:**

The development of information and psychological stability by increasing tolerance to uncertainty is a perspective area of research in the field of mental protection of the population of Ukraine.

**Disclosure of Interest:**

None Declared

